# Recruitment constraints in Singapore's fluted giant clam (*Tridacna squamosa*) population—A dispersal model approach

**DOI:** 10.1371/journal.pone.0058819

**Published:** 2013-03-13

**Authors:** Mei Lin Neo, Paul L. A. Erftemeijer, Jan K. L. van Beek, Dirk S. van Maren, Serena L-M. Teo, Peter A. Todd

**Affiliations:** 1 Experimental Marine Ecology Laboratory, Department of Biological Sciences, National University of Singapore, Singapore, Singapore; 2 Tropical Marine Science Institute, National University of Singapore, Singapore, Singapore; 3 Sinclair Knight Merz (SKM), Perth, Australia; 4 The UWA Oceans Institute, University of Western Australia, Crawley, Australia; 5 Deltares, (formerly Delft Hydraulics), Delft, The Netherlands; Institute of Marine Research, Norway

## Abstract

Recruitment constraints on Singapore's dwindling fluted giant clam, *Tridacna squamosa*, population were studied by modelling fertilisation, larval transport, and settlement using real-time hydrodynamic forcing combined with knowledge of spawning characteristics, larval development, behaviour, and settlement cues. Larval transport was simulated using a finite-volume advection-diffusion model coupled to a three-dimensional hydrodynamic model. Three recruitment constraint hypotheses were tested: 1) there is limited connectivity between Singapore's reefs and other reefs in the region, 2) there is limited exchange within Singapore's Southern Islands, and 3) there exist low-density constraints to fertilisation efficacy (component Allee effects). Results showed that connectivity among giant clam populations was primarily determined by residual hydrodynamic flows and spawning time, with greatest chances of successful settlement occurring when spawning and subsequent larval dispersal coincided with the period of lowest residual flow. Simulations suggested poor larval transport from reefs located along the Peninsular Malaysia to Singapore, probably due to strong surface currents between the Andaman Sea and South China Sea combined with a major land barrier disrupting larval movement among reefs. The model, however, predicted offshore coral reefs to the southeast of Singapore (Bintan and Batam) may represent a significant source of larvae. Larval exchange within Singapore's Southern Islands varied substantially depending on the locations of source and sink reefs as well as spawning time; but all simulations resulted in low settler densities (2.1–68.6 settled individuals per 10,000 m^2^). Poor fertilisation rates predicted by the model indicate that the low density and scattered distribution of the remaining *T. squamosa* in Singapore are likely to significantly inhibit any natural recovery of local stocks.

## Introduction

Giant clam populations in Singapore have declined since the early 1950s due to overharvesting and the loss of coral reef habitats [1], [2]. Surveys of Singapore's Southern Islands conducted in 2009/2010 indicate that only a very small adult population of two species (*Tridacna crocea* and *T. squamosa*) persists, while *Hippopus hippopus*, *T. giga* and *T. maxima*, which used to be present, are now locally extinct [2], [3]. All the clams surveyed were mature [3], indicating a lack of local recruitment and possibly a low chance of natural recovery. For giant clam populations to remain viable, each reproducing clam must replace itself within a generation length. This encompasses the probability that: 1) broadcast gametes meet and fertilise, 2) larvae are dispersed, settle successfully and grow, and 3) the new clams reach reproductive age and produce new larvae. Singapore's giant clam populations are probably constrained by component Allee effects, i.e. their low densities reduce the likelihood of successful fertilisation and subsequent recruitment [4], [5]. As populations of marine organisms were thought to be ‘open’ with large effective population sizes [6], Allee effects were rarely considered important [7]. However, broadcast spawning marine species experiencing reduced populations, due to over-exploitation for example, are now believed to be susceptible to Allee effects [7], [8].

Giant clams are broadcast spawners with high fecundity but poor early life survivorship [9]. Published recruitment studies of giant clams are few in number [10], [11], and none address larval dispersal mechanisms despite the well-documented importance of larval transport for many marine invertebrate species [12], [13]. With a planktonic phase of approximately nine days [14], their larvae are likely to have a substantial dispersal capability (as larvae can potentially be transported hundreds of kilometres in that timeframe), which may facilitate connectivity among populations [15], [16]. Conversely, results from giant clam genetic studies have indicated restricted gene flow, suggesting lower levels of exchange [17], [18]. Ocean current patterns have been invoked to explain such genetic divergences among marine invertebrate populations [19], as they can influence temporal and spatial physical processes that potentially restrict larval dispersal and gene flow [20], [21].

Efforts to conserve giant clams in Singapore are underway [2], [22] with baseline research conducted on their distribution [1], [3], autecology [23], [24], [25] and behaviour [26], [27]. Regionally, studies on depleted giant clam populations have examined the fundamental genetic structures of broodstock populations vis-á-vis enhancing genetic diversity in progeny batches [28], [29] and reintroducing captive-reared clams onto reefs [30]. Larval life stages are important considerations when rebuilding marine invertebrate stocks [31], especially as recruitment rates and population connectivity for molluscs are dependent on the dispersal patterns of planktonic larvae from spawning areas to settlement grounds [32]. Knowledge on larval dispersal within Singapore waters is essential to ensure a sustainable population of giant clams, for instance, by helping to identify nursery sites that have the greatest potential as a source of larvae for other Southern Islands reefs. Through a combination of hydrodynamic and behavioural modelling of clam larvae, the present study simulates connectivity and recruitment to investigate potential constraints on the transport success of fluted giant clam, *T. squamosa* larvae—expressed as the number of larvae assumed to have settled onto local reefs by the end of their pelagic cycle. We tested three hypotheses: 1) there is limited connectivity between Singapore's reefs and other reefs in the region, 2) there is limited exchange within Singapore's Southern Islands and 3) there exist low-density constraints to fertilisation efficacy (component Allee effects).

## Materials and Methods

Egg and larval transport was modelled using a three-dimensional (3D) hydrodynamic model and an Eulerian transport model coupled with mathematical definitions of larval characteristics, including estimates of sedimentation velocity, growth, behaviour and development of giant clam larvae.

### Hydrodynamic model

Delft3D is a modelling system that allows the simulation of flow, wave, sediment transport, and ecological processes (see [33], [34]). By solving well-established shallow-water hydrostatic pressure equations, Delft3D-FLOW can simulate the 3D unsteady flow and transport phenomena resulting from tidal and meteorological forcing [34], [35]. These model equations, formulated in orthogonal curvilinear coordinates, are discretised onto a staggered Arakawa-C grid and time-integrated by means of an alternating direction implicit (ADI) numerical scheme in horizontal directions and by the Crank-Nicolson method along the vertical, which is either discretised by terrain following coordinates (σ-transformation) or through horizontal z-layers [36]. The solution is mass conserving at every grid cell and time step. This code is extended with transport of salt and heat content and with four turbulence models such as the k-ε model [37] for vertical exchange of horizontal momentum and matter or heat, possibly subjected to density stratification, and with other models for lateral mixing. Along the open sea boundaries, tidal harmonics for water level or currents and concentration patterns for constituents are imposed. The computed flow and mass-transport patterns can be coupled off-line to other Delft3D modules, such as the Eulerian advection-diffusion model Delft3D-WAQ (see below). In this off-line coupling, aggregation in time step and/or grid cells is optional for speeding up subsequent analyses. A number of studies [e.g. 33], [38] have demonstrated the applicability of Delft3D to the modelling of shallow-water hydrodynamics.

### Model grid resolution, water layers, and model forcing

**Figure 1:**
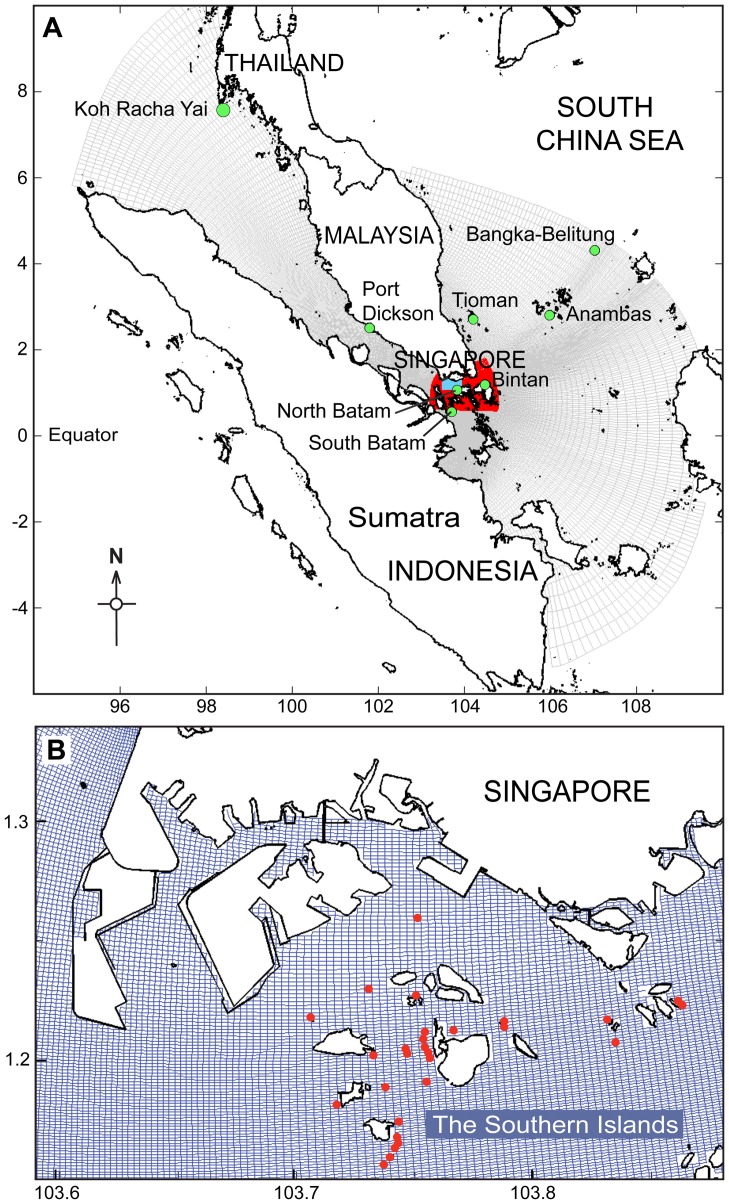
Singapore regional model. This model is composed of 3 domains. A) The overall outer domain including Peninsular Malaysia and the 8 regional release points (green dots). The red and blue domains represent the refined grid resolutions for Singapore's coastal waters. B) The blue grid encompasses the waters surrounding Singapore's Southern Islands. The red dots represent the 28 release points (i.e. the positions of *T. squamosa* in Singapore).

Here we used a locally refined version [39] of the Singapore Regional Model (see [40]). This model is composed of three domains [41]. The model's outer domain has a grid cell size decreasing from 30 km near the boundaries to ∼300 m around Singapore (see Figure 1). The middle domain (in red) has the same resolution (300 m), but the local domain (in blue) around Singapore's islands are refined by a factor three compared to the outer and middle domains, leading to grid cell sizes down to 100 m.

The model was forced at its three open boundaries (the Andaman Sea in the northwest, the South China Sea in the northeast, and the Java Sea in the southeast) by 8 tidal constituents and a mean annual cycle of the monsoon-induced water level, derived from 15 years of Topex-Poseidon and Jason-1 satellite altimetry (see [40], [42]).

### Calibration and validation of hydrodynamics

The hydrodynamics in Singapore coastal waters are complex, with predominantly semi-diurnal water level variations but diurnal currents. Superimposed on this are compound tides generated by semi-diurnal and diurnal constituents with a periodicity equal to the spring neap cycle (approximately 2 weeks), and monsoon currents [39]. Within Singapore's Southern Islands area, dominant flow is eastward from April/May to September/October, and westward during the other months. This seasonal variation, and the two-weekly variations, is well reproduced by the model. It should be noted that the stations Banyan and Sawa are within the Southern Islands area, where large-scale clockwise circulation generates more pronounced eastward currents than in the open Singapore Strait south of the islands [41]. Therefore residual currents within the Southern Islands group in April tend to be directed eastward while in the open strait they may be directed westward.

### Transport model

Transport of giant clam eggs and larvae was modelled using the water quality module of Delft3D (Delft3D-WAQ) [43]. Delft3D-WAQ is a transport model that has been successfully applied to dispersal simulations of seagrass seeds, fish larvae and mangrove propagules [13], [44], [45], [46]. The model calculates the concentrations of ‘substances’ (in this case: either eggs or larvae) for each time-step as a function of the initial concentrations, advective and dispersive transport, and biological characteristics and processes. Delft3D-WAQ is an Eulerian model based on the finite-volume method (i.e. multiplication of fluxes with concentrations to obtain masses across internal and external boundaries). Both finite-volume methods and particle tracking model approaches can (in principle) provide comparable results [47]. With our focus on mid-field and far-field effects, the WAQ model (including the extensive and well-validated biological process library) is more appropriate than a particle-tracking method. The main advantages of particle-tracking are that it offers sub-grid model resolution as well as the opportunity to track individual seedlings, both of which are not very relevant to our study. The actual water system is represented within Delft3D-WAQ by means of computational elements (segments). The flow between segments is derived from the hydrodynamic model (Delft3D-FLOW) of the same resolution (i.e. down to 100 m around the Southern Islands).

### Definition of processes and parameters

Specific release points outside of Singapore (8 points) (Figure 1A) and within the Southern Islands (28 points) (Figure 1B) were selected as initial spawning points for modelling the transport of eggs and larvae. Factors that are known to affect larval growth and development were incorporated into the transport model: spawning periods, different stages of larval development (with different behavioural rules), larval swimming behaviour and mortality of larvae at respective stages. The details of larval stages, specific behavioural rules, processes and parameters incorporated into the model are described below.

### Spawning

Spawning seasonality in *T. squamosa* varies among localities [4], [48], [49] but mature gametes can generally be found throughout most of the year [50]. Since the actual spawning periods in Singapore are unknown, three time points representing local seasonality were selected to investigate the effects of spawning times on recruitment success. Spawning in giant clams often occurs during full moon or new moon [51], [52] and this was therefore taken into account with the transport of either eggs or larvae modelled assuming each simulation was a single spawning event on the following lunar periods: 22 January (new moon), 10 April (full moon) and 18 June (new moon) 2004. Giant clams are benthic spawners, hence all eggs were released in the lowest 10% of the model layer representing the water column.

### Development and behaviour of eggs and larvae

In the model, five developmental stages [25] were distinguished based on their behavioural and physical traits in relation to horizontal and vertical transport.

Stage 1: Passive horizontal pelagic transport of eggs homogenously distributed within the water column. At day 0, eggs were assumed to have neutral buoyancy while being passively transported by currents.

Stage 2: Passive horizontal pelagic transport of trochophores as in Stage 1. Assuming all the released eggs were fertilised, upon hatching after 24 hours, the trochophores have limited overall locomotion [53] and are largely transported by currents. With their poor swimming ability, vertical transport with diel migration is limited at this stage (see “Sensitivity analyses” below). The distinction between eggs and pelagic trochophores was made to facilitate growth parameter settings such as mortality rates and sedimentation velocity.

Stage 3: Passive horizontal pelagic transport of veliger larvae. Locomotion of early veligers (2 to 4 days old) is primarily through ciliary band movement [54], [55], which affects vertical position but is negligible in the horizontal dimension compared to the strength of the currents. Therefore, only vertical movement was simulated in the model, by varying the larvae's sedimentation velocity (see “Sensitivity analyses” below). Stage 3 mortality rates and sedimentation velocity were different to those in Stage 2.

Stage 4: Passive horizontal pelagic transport of veliger larvae. In Stage 4 (5 to 7 days old), late veligers develop a primitive foot—an initiation of their sedentary lifestyle, but still rely on swimming to move between the surface water and bottom layers. The sedentary component of Stage 4 distinguishes it from Stage 3.

**Figure 2:**
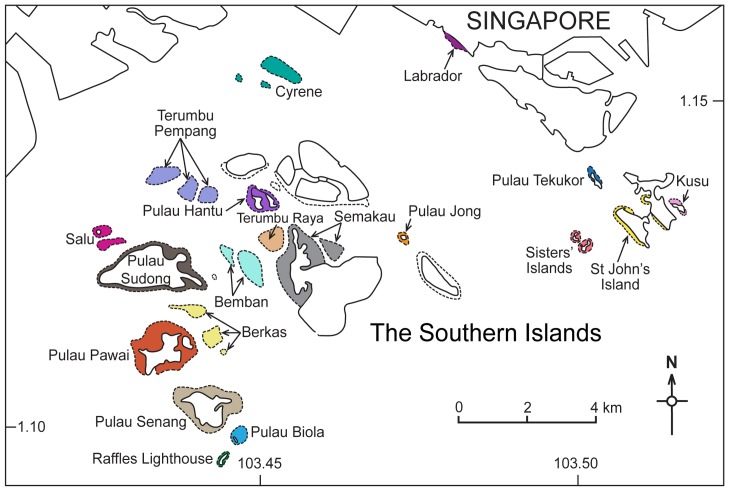
Singapore's Southern Islands. Coral reef areas (in colour) among Singapore's Southern Islands used to estimate transport success. Each colour corresponds to a distinct potential sink site.

Stage 5: During the last metamorphosis stage, the velum and fully developed foot of pediveligers allows them to alternately swim and crawl on the benthos; over time, these larvae become increasingly sedentary [11], [56]. Transport is completed after this metamorphosis stage. In Stage 5, juveniles (8 to 9 days old) either continue to exhibit the behaviour of Stage 4 larvae, or settle onto the coral reefs. Giant clam larvae respond to settlement cues such as the presence of crustose coralline algae [11] and/or conspecific adults [57], [58], both of which are found on coral reefs. Hence, in our model, larval settlement was mimicked when larvae passed over coral reef areas (see Figure 2).

Growth parameters of the various stages were estimated using existing data obtained from laboratory experiments [25] and mariculture literature [53], [59], [60]. The average values for concentrations of egg release and development rates for each stage were chosen as default model settings (see “Sensitivity analyses” below). For each dispersal scenario, the transport model was run for a period of 15 days [52] as previous work indicated this was the time during which *T. squamosa* larval settlement occurs (unpublished data).

### Sensitivity analyses

Four sensitivity scenarios (and the default scenario) were performed using a single release site on Pulau Semakau (1°12′10.30″N, 103°45′25.45″E). Three parameters were examined for their effect on larval transport success: seasonality, larval sedimentation velocity incorporating diel vertical migration (i.e. positive in the night and negative in the day), and mortality rates. For all scenarios, 3 time points were chosen: 22 January, 10 April and 18 June 2004 (as described earlier). Approximated settling velocities were varied between the larval stages [54]. Across all scenarios, Stage 1 (eggs) was assumed neutral buoyancy (0 cm s^−1^). Three sedimentation velocity scenarios were set for larvae, where a) all larval stages assumed settling velocities of 0 cm s^−1^ (*Neutral*), b) all larval stages assumed average settling velocities (+/−0.0579 cm s^−1^; *Average*) and c) Stage 1 = 0 cm s^−1^; Stage 2 = +/−0.0579 cm s^−1^ and Stages 3–5 = +/−0.1 cm s^−1^ (*Default*). Input values for settling rates were obtained from bivalve larvae literature [54], [61] following a diel vertical migration [62]. Three scenarios were set to test effects of mortality rates on survivorship, where all larval stages experienced a) lowest mortality (*Low mortality*), b) highest mortality (*High mortality*) and c) average mortality (*Default*). For the respective larval stages, mortality rates were estimated using published data of other giant clam species (Table 1). The settlers' distribution patterns were analysed using graphic contour plots that indicated both the temporal and spatial distribution of larvae (densities m^−2^). At each time point, the number of settlers (i.e. total bottom larvae) that had arrived on all of the local coral reefs at the end of the model run was summed to calculate the transport success for the respective scenarios.

**Table 1:** Mortality rates for *Tridacna* larvae.

Model	Source(s)	Stage duration (*D*) according to [52]	Scenarios for sensitivity analyses					
			Low mortality rates		High mortality rates		Default model settings	
			p_m_	*k*	p_m_	*k*	p_m_	*k*
Stage 1 (eggs)	-	0 h	0	0	0	0	0	0
Stage 2 (trochophores)	[63]	24 h	0.167	0.183	0.933	2.703	0.567	0.836
Stages 3 (D-veliger)	[63], [64]	48 h	0.200	0.112	0.945	1.450	0.529	0.376
Stage 4 (late veliger)	[63], [65]	48 h	0.200	0.112	0.945	1.450	0.529	0.376
Stage 5 (pediveliger)	[63]	96 h	0.571	0.212	0.950	0.749	0.816	0.423

Where data for *Tridacna squamosa* were deficient, larval mortality at 5 larval stages was extrapolated from published and unpublished reports of other giant clam species. Data have been reworked to fit into the model, *k* = −In(1−p_m_)/(*D*/24) in which *D* is stage duration and p_m_ is the proportion of dead larvae.

### Modelling scenarios

*Tridacna squamosa* have a high fecundity, releasing eggs of 420,000 to 46,000,000 eggs released per individual each spawning [25], [49]. In the model, a fixed average initial concentration of 4,500,000 eggs was released over a 15-minute time step. Based on the sensitivity analyses, *Default* settings were used for all transport models. Three main scenarios were considered in the investigation of larval connectivity and the effects of hydrodynamics on larval recruitment.

1. Dispersal patterns from regional donor reefs to Singapore—this scenario examined the potential of regional coral reefs to donate giant clam larvae to reefs in Singapore (i.e. recipient reefs), modelled using the hydrodynamics simulated for 22 January, 10 April and 18 June 2004 over a period of 15 days of transport. Eight release points, i.e. possible donor sites, were examined individually (8 separate runs): Koh Racha Yai (Thailand), Port Dickson (Malaysia), north and south Batam, Bintan, Bangka-Belitung and Anambas (all Indonesia), and Tioman Island (Malaysia) (see Figure 1A for exact localities).
2. Dispersal patterns within Southern Islands, Singapore—this scenario examined source-sink dynamics via larval dispersal within the Southern Islands reefs, modelled using the hydrodynamics simulated for 10 April 2004 over a period of 15 days of transport. Transport model was performed in April 2004 based on the mass coral spawning in Singapore [66], assuming that it was an ‘ideal’ period for larval dispersal. For this study, source reefs are habitats optimal for restocking while sink reefs are habitats where restocking is likely to be fruitless, but can serve as locations for the recruitment of larvae via source reefs [67]. To identify respective source and sink reefs within the Southern Islands, reefs supporting the current *T. squamosa* population (n = 28) in Singapore [3] were individually examined as possible sources of larvae in this scenario. Release points were as follows: Raffles Lighthouse 01–02, Biola 01–03, Senang, Pawai, Berkas, Sudong, Salu, Beting Bemban Besar 01–02, Terumbu Raya, Semakau 01–05, Terumbu Semakau, Jong 01–02, Terumbu Pempang Tengah, Hantu, Sisters 01–02, Kusu 01–02 and Cyrene.
3. Egg dispersal potential—this scenario examined egg dispersal movement within the Southern Islands reefs; modelled using the hydrodynamics simulated for 10 April 2004 over a period of 6 hours. As egg masses are known triggers for eliciting a spawning response (resulting in either release of sperm or eggs) in adult clams [68], [69], transport of eggs was of greatest interest. Release points represented the current *T. squamosa* population (as described earlier) and eggs were released at each location (28 separate runs).

### Analysis of outputs from modelled scenarios

To quantify larval transport patterns and concentrations, post-model processing was carried out to calculate the following output parameters:

1. Dispersal patterns from regional donor reefs to Singapore—at each time point, the percentage of successful settlers that had arrived on Singapore's coral reefs at the end of the model run was summed to calculate transport success from respective donor locations.
2. Dispersal patterns within Southern Islands, Singapore—the density of successful settlers (i.e. number of larvae per 10,000 m^2^) that had arrived on the local coral reefs was computed at the end of the model run. The model grid area was subdivided into 19 reef sections (Figure 2), delimited by the 20 m-depth contour. For each section, the number of larvae per compartment was summed to determine the transport success.
3. Egg dispersal potential—time-series plots describing the arrival time of eggs over certain clams was determined by plotting larval density (number per m^2^) in the model at each observation point (usually one grid cell) showing the accumulation of eggs over any specified coral reef area. Donor-recipient clams were identified with the following parameters: distance between clam pairs, arrival time of eggs, and peak number of eggs arrived per m^2^.

## Results

### Sensitivity analyses

**Figure 3:**
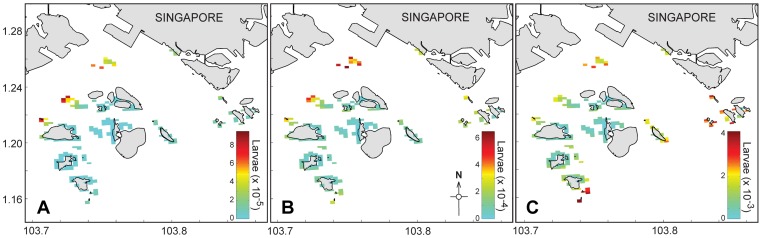
Contour plots of settler density. Distribution patterns of giant clam larvae on local coral reefs at the end of transport phase for the three spawning periods: A) 22 January, B) 10 April and C) 18 June 2004.

**Figure 4:**
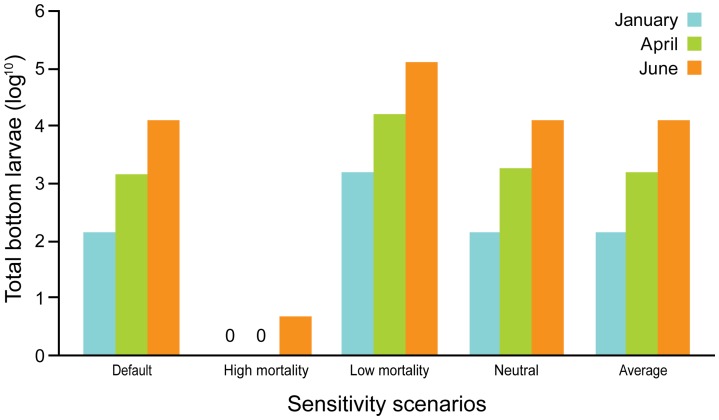
Sensitivity scenario analyses. Sensitivity testing on the effect of mortality and sedimentation velocity settings on numbers of settled larvae for three different timings of release (January, April, June).

Sensitivity analyses of the release times indicated that successful settlement of giant clam larvae on Singapore's reefs could potentially be achieved throughout the year, with the greatest chances of successful larval settlement when gametes were released during June. Density of larval settlement on reefs increased over the months: January<April<June (Figure 3). January is the period with the greatest westward flow velocity whereas eastward flow peaks in June–July (with April being the transitional period); settlement is therefore expected to decrease again after June–July. Settlement success varied among islands, where in January and April, the northwestern reefs had higher densities of settled larvae, while northern and southern reefs had higher densities of settled larvae in June (Figure 3). Variations in the larval sedimentation velocity, following a diel vertical migration pattern, did not affect larval transport success (Figure 4). However, mortality rates for each larval stage had a significant effect on transport success. Highest mortality rates (see Table 1) resulted in almost no larval settlement on the reefs (Figure 4).

### Regional donor reefs and Singapore

Transport successes of larvae to Singapore from five donor localities in neighbouring countries (Koh Racha Yai, Port Dickson, Bangka-Belitung, Tioman Island and Anambas) were very poor (∼0%) (Table 2). Three other donor localities (north and south Batam, and Bintan) had more positive transport success. Larvae from north Batam had the highest settlement success of 61.58% on Singapore's reefs in June, while Bintan had high settlement success throughout the year (January: 30.86%, April: 44.53%, June: 19.40%) (Table 2).

**Table 2:** Proportion of larvae settled onto Singapore's coral reefs.

Donor coral reefs	Transport success (%)		
	January 2004	April 2004	June 2004
Koh Racha Yai	0.00	0.00	0.00
Port Dickson	0.00	0.00	0.00
South Batam	0.01	0.80	0.51
North Batam	5.94	22.50	61.58
Bintan	30.86	44.53	19.40
Bangka-Belitung	0.00	0.00	0.00
Tioman	0.13	0.00	0.00
Anambas	0.00	0.00	0.00

Percent of total number of *T. squamosa* larvae released from various regional donor reefs that reached recipient reefs around Singapore's Southern Islands.

### Southern Islands reefs, Singapore

A summation matrix of total bottom larvae was produced to identify the prospective source and sink sites on Southern Islands reefs for analysis of local reef connectivity. Assuming all 19 sections were potential sink sites, larval transport success (per 10,000 m^2^ of reef area) was low among Southern Islands reefs (Table 3). The eastern islands, such as Sisters' and Kusu islands (Figure 2), could be potential source reefs as, when larvae were released from these locations, surrounding reefs were able to receive high numbers of settled larvae per 10,000 m^2^ (Table 3). Four most potential sink sites were identified: Cyrene, Tekukor, Raffles Lighthouse and Salu, where from a single source site (Sisters 02) each of the mentioned reefs received 68.6, 50.2, 46.2 and 38.8 settled larvae per 10,000 m^2^ respectively (Table 3). Coral reefs found within the central area, such as Pulau Hantu, Semakau, Pulau Sudong (Figure 2), were generally poor or moderate sources and/or sinks, with the majority of sites receiving fewer than 20 larvae per 10,000 m^2^.

**Table 3:** Source-sink dynamics for Singapore's coral reefs.

Source sites	Potential sink sites																		
	Raffles Lighthouse	Pulau Biola	Pulau Senang	Pulau Pawai	Berkas	Pulau Sudong	Bemban	Salu	Terumbu Raya	Semakau	Pulau Jong	Sisters' islands	St John's island	Pulau Hantu	Terumbu Pempang	Kusu	Pulau Tekukor	Cyrene	Labrador
Raffles Lighthouse 01	18.9	13.2	5.4	4.3	7.2	5.3	3.9	19.6	3.6	2.2	11.2	14.2	11.6	7.2	17.6	3.1	21.6	33.9	17.7
Raffles Lighthouse 02	17.9	12.5	5.1	4.0	6.8	5.0	3.7	18.5	3.4	2.1	10.6	13.5	11.0	6.9	16.7	2.9	20.4	32.1	16.7
Biola 01	20.1	14.0	5.7	4.5	7.6	5.5	4.1	20.5	3.8	2.3	11.8	15.0	12.3	7.6	18.5	3.3	22.7	35.5	18.6
Biola 02	21.4	14.9	6.1	4.7	8.0	5.8	4.3	21.6	4.0	2.5	12.5	15.9	13.0	8.0	19.3	3.5	24.0	37.4	19.6
Biola 03	21.3	14.8	6.0	4.7	8.0	5.8	4.3	21.5	4.0	2.5	12.4	15.8	12.9	8.0	19.3	3.5	23.9	37.2	19.6
Senang	23.2	16.2	6.6	5.1	8.7	6.4	4.7	23.5	4.3	2.7	13.7	17.4	14.2	8.7	21.1	3.9	26.3	40.7	21.5
Pawai	22.6	15.8	6.5	5.0	8.5	6.2	4.6	23.0	4.2	2.6	13.3	17.0	13.9	8.5	20.6	3.8	25.7	39.9	21.0
Berkas	22.9	16.3	6.7	5.3	9.1	6.8	4.9	25.3	4.6	2.8	14.4	18.6	15.2	9.4	23.0	4.1	28.4	44.7	23.6
Sudong	21.1	15.1	6.3	5.1	8.6	6.5	4.7	24.5	4.4	2.7	13.7	17.7	14.5	9.1	22.3	3.8	27.1	43.4	22.8
Salu	18.8	13.7	5.8	4.8	8.1	6.3	4.4	23.9	4.2	2.5	12.9	16.6	13.6	8.8	21.9	3.4	25.8	42.5	21.9
Beting Bemban Besar 01	26.6	18.8	7.7	6.0	10.3	7.5	5.5	27.9	5.2	3.2	16.4	21.2	17.4	10.4	25.2	4.9	32.1	49.3	26.5
Beting Bemban Besar 02	26.6	18.8	7.7	6.0	10.3	7.5	5.5	27.9	5.2	3.2	16.4	21.1	17.3	10.4	25.2	4.9	32.1	49.3	26.4
Terumbu Raya	25.2	17.9	7.4	5.8	9.9	7.3	5.4	27.3	5.0	3.1	15.9	20.5	16.8	10.2	24.7	4.7	31.2	48.2	25.9
Semakau 01	27.2	19.2	7.8	6.1	10.5	7.6	5.6	28.3	5.2	3.2	16.7	21.5	17.7	10.5	25.5	5.0	32.6	49.8	26.9
Semakau 02	25.1	17.8	7.3	5.8	9.8	7.3	5.3	27.0	5.0	3.1	15.7	20.3	16.6	10.1	24.4	4.6	30.9	47.8	25.5
Semakau 03	26.8	18.9	7.7	6.0	10.3	7.6	5.6	28.0	5.2	3.2	16.5	21.2	17.4	10.4	25.2	4.9	32.2	49.5	26.5
Semakau 04	26.3	18.6	7.6	6.0	10.2	7.5	5.5	27.7	5.1	3.2	16.3	21.0	17.2	10.3	25.1	4.8	31.9	48.9	26.3
Semakau 05	26.6	18.8	7.7	6.0	10.3	7.5	5.5	27.8	5.2	3.2	16.3	21.1	17.3	10.4	25.2	4.8	32.0	49.3	26.4
Terumbu Semakau	24.8	17.7	7.3	5.8	9.8	7.3	5.3	27.1	5.0	3.0	15.7	20.3	16.6	10.1	24.5	4.6	30.9	48.0	25.7
Jong 01	28.3	20.1	8.2	6.4	11.0	8.0	5.9	29.7	5.5	3.4	17.7	23.0	18.9	11.1	26.8	5.5	35.0	52.7	28.9
Jong 02	28.3	20.1	8.2	6.4	11.0	8.0	5.9	29.7	5.5	3.4	17.7	23.0	18.9	11.1	26.8	5.5	35.0	52.7	28.9
Terumbu Pempang Tengah	22.6	16.2	6.7	5.4	9.1	6.9	4.9	25.7	4.6	2.8	14.6	18.8	15.4	9.5	23.3	4.1	28.8	45.6	24.2
Hantu	23.2	16.6	6.9	5.5	9.3	7.0	5.1	26.2	4.8	2.9	14.9	19.3	15.8	9.7	23.8	4.3	29.6	46.5	24.7
Sisters 01	31.4	22.2	9.0	6.9	12.0	8.5	6.4	31.4	5.9	3.7	19.4	25.2	20.8	11.9	28.4	6.2	37.9	56.0	31.1
Sisters 02	46.2	32.1	12.5	9.2	16.2	10.8	8.5	38.8	7.7	5.0	26.6	34.6	28.5	14.9	34.5	9.7	50.2	68.6	39.6
Kusu 01	45.6	31.7	12.4	9.1	16.0	10.7	8.4	38.1	7.5	4.9	26.4	34.5	28.5	14.7	34.0	9.8	49.9	67.8	39.2
Kusu 02	46.5	32.4	12.6	9.2	16.3	10.7	8.5	38.6	7.7	5.0	26.9	35.1	29.0	14.9	34.4	10.1	50.7	68.5	39.9
Cyrene	21.4	15.6	6.5	5.4	9.1	7.0	4.9	26.6	4.7	2.8	14.6	19.0	15.6	9.8	24.3	4.0	29.6	48.0	25.4

Summation matrix of settled larvae (per 10,000 m^2^) showing the potential sources (rows) versus sinks (column) among the Southern Islands coral reefs. Source sites are arranged according to the descending shortest straight-line distance to the mainland.

### Egg dispersal potential

To assess low-density constraints to fertilisation efficacy, dispersal potential of giant clam eggs between donor and recipient clams within known Singapore localities was analysed from the point of release (0 hours) to 6 hours later (estimated viability of eggs; unpublished data). Connectivity between *T. squamosa* individuals was limited to either the dense clusters of >2 clams (Raffles Lighthouse and Biola, Beting Bemban Besar and Semakau) or paired clam individuals that were in close proximity (within Jong and within Kusu) (Table 4). Based on the results, for eggs to arrive over their nearest-neighbour clams within the period of their viability, clams must be within a vicinity of no more than 2000 m. However, the number of eggs arriving at recipient clams varied across sites, regardless of time or distance (Table 4).

**Table 4:** Egg dispersal potential of individual giant clams among the Southern Islands reefs.

Donor clam	Recipient clam	Distance (m)	Time taken for most eggs to arrive at clam (h)	Peak number of eggs per m^2^
≤5 eggs per m^2^				
Raffles Lighthouse 01	Raffles Lighthouse 02	189.80	00:15	1.92
Biola 01	Raffles Lighthouse 02	362.28	00:15	1.52
Biola 02	Biola 01	340.43	00:15	3.73
Biola 03	Biola 01	240.21	00:15	4.29
Beting Bemban Besar 01	Beting Bemban Besar 02	153.23	00:45	3.35
	Semakau 04	992.57	01:00	2.80
	Semakau 05	850.08	01:00	2.45
	Semakau 03	1126.64	01:30	1.15
	Semakau 02	1598.75	02:15	2.22
Beting Bemban Besar 02	Semakau 04	941.07	00:45	1.82
	Semakau 05	834.62	01:00	1.35
	Semakau 02	1499.68	02:15	2.23
Semakau 01	Semakau 05	410.25	01:00	2.14
	Semakau 04	626.81	01:15	1.70
	Semakau 03	860.57	01:30	4.26
	Semakau 02	1488.08	02:00	1.24
Semakau 03	Semakau 02	632.81	01:30	1.20
Semakau 04	Semakau 03	227.33	00:30	2.89
	Semakau 02	873.49	01:30	4.57
Semakau 05	Semakau 03	471.36	00:45	3.57
	Semakau 02	1104.78	01:45	3.86
Hantu	Terumbu Pempang Tengah	2228.47	01:45	1.80
5≤eggs≤10 per m^2^				
Biola 02	Biola 03	115.88	00:15	6.02
Jong 02	Jong 01	172.94	00:15	9.66
≥10 eggs per m^2^				
Biola 03	Biola 02	115.88	00:15	11.58
Beting Bemban Besar 02	Beting Bemban Besar 01	153.23	00:15	12.99
Semakau 05	Semakau 04	248.52	00:15	18.88
Kusu 02	Kusu 01	267.79	00:15	10.00

Only clams with more than one egg per m^2^ arriving onto a reef within the first 6 hours were considered to constitute successful transport.

## Discussion

For many sessile marine invertebrates, planktonic stages are the only mode of dispersal. These stages facilitate their widespread distribution [12], [70], re-colonisation of areas after local extirpation [71], and promote gene flow [72]. Here, we present the first modelling study that examines the transport and recruitment of fluted giant clam larvae from outside and within Singapore waters using real-time hydrodynamics forcing and incorporating larval behavioural processes. Our findings suggest that larval connectivity among reefs is largely dependent on monsoons that influence larval transport and settlement through the direction and strength of residual currents. Potential larval donor reefs in the region appear to be largely restricted to the south of Singapore (Batam and Bintan). The sheltering effect of land barriers probably affects input from other neighbouring countries. Egg dispersal and local recruitment to the existing *T. squamosa* population was found to be limited in our model simulations, indicating poor reproductive efficacy. Hence, the fluted giant clam population in Singapore is constrained by component Allee effects [7], [8], that is, numbers of remaining clams are too few and sparsely distributed, leading to low fertilisation success.

Giant clam larval transport success appears to be largely driven by variability in annual hydrodynamics (for the year that was modelled). Consistent westward residual currents in the outer straits of Singapore during January and in April drive larval transport towards the west, with higher larval retention in the northwestern reefs. In contrast, the lack of residuals in June allows much higher retention in the northern and southern reefs with higher larval settlement. In Singapore, broadcasting corals annually spawn in late March or mid April [66]. While the moderate residuals during this time may be favourable for coral larvae with short settlement periods [73] those with longer life cycles, such as giant clams, may experience dilution of larvae into the outer straits when released during this period. The near absence of residual currents in June favours retention of clam larvae, reducing offshore dispersal. Larval mortality also greatly influences transport success, which in turn affects juvenile recruitment on reefs [60]. Sedimentation velocity and diel vertical migration, however, have negligible effects on transport success, suggesting that ocean currents primarily influence larval dispersal [74]. Results from this modelling study should be interpreted with caution, bearing in mind the various assumptions made. The transport success and dispersal distances predicted by the model probably do not equate to actual recruitment success in the field.

The poor larval connectivity from regional reefs to Singapore could be explained by the strong surface currents flowing between the Andaman Sea and South China Sea during the monsoons [75] that move larvae out of the Singapore Strait with little retention. Poor larval connectivity with most external potential donor reefs may also be attributed to Peninsular Malaysia. Phylogeographic studies of marine invertebrates and mangroves have shown that this peninsular acts as a barrier that disrupts gene flows between the east and west coasts, corresponding to the western Sunda Shelf Barrier [18], [19], [76]. Population genetic breaks in *T. crocea* populations on the Sunda Shelf and western Indonesia also provide evidence for limited connectivity in this region [17], [18]. In contrast, offshore coral reefs located to the southeast of Singapore, combined with the favourable westward residuals along the straits [77] and absence of significant land barriers, encourage high larval settlement and retention. As predicted by the model, *T. squamosa* populations in Batam and Bintan could provide a significant stock of source larvae for the clam-depauperate reefs in Singapore waters; possibly facilitating the natural recovery of populations.

Our model results indicate that source larvae from Singapore's eastern islands settle in higher numbers on the western reefs within the Southern Islands. This observation could be explained by the westward current residuals throughout the year [77], favouring larval transport in a westward direction. The Southern Islands reefs can potentially receive larvae from any of the local 28 reefs that currently host giant clams and such connectivity was identified in Singapore's *T. squamosa* population via genetic analysis [3]. Reefs on the northernmost (Cyrene and Pulau Tekukor) and southernmost (Raffles Lighthouse) reaches of the Southern Islands received most larvae per unit area in the model, with fewest larvae per unit area settling among the central island clusters (Semakau and Sudong). These larval dispersal patterns may be influenced by the fine-scale tidal flows within the Southern Islands area [77], [78] and the presence of land barriers [76], influencing the source-sink dynamics. For example, sheltered reefs off Semakau and Sudong exhibited much lower settler densities compared to the more exposed reefs off Cyrene and Pulau Tekukor. Singapore's healthiest reef, Raffles Lighthouse [79] is, perhaps surprisingly, not the best sink. The reefs at Raffles Lighthouse experience a larger tidal range due to their proximity to the Singapore Straits [78] and there are no nearby islands to the east, west or south, thus larvae may easily be transported away. Cyrene, on the other hand, is protected by surrounding land masses [41], [80], leading to higher larval retention.

Fertilisation success in giant clams can be measured by the eggs' dispersal potential since a known chemical trigger for spawning synchrony and sperm release among clams depends on the presence of eggs [68], [69]. For successful fertilisation of gametes, giant clams need to be within close proximity (ideally, aggregated) [26] for the detection of chemical cues from egg masses released by neighbouring individuals. For the 28 *T. squamosa* remaining in Singapore waters, our model showed limited potential for egg masses to be dispersed towards/over neighbouring clams within the period of egg viability. This limited connectivity between individuals may partially explain the absence of juvenile fluted giant clams on local reefs [3]. The model results revealed that only clams found on the same reefs could potentially trigger spawning and result in subsequent fertilisation. Previous modelling studies have suggested that, even with small nearest-neighbour distances, the percentage of eggs fertilised can be limited—especially under high turbulence conditions such as in the surf zone [81]. Field data from [82] showed that, even with high densities of mature giant clams on Rose Atoll, recruitment was low. Fertilisation efficiency is further known to vary with species and environment [69], [83]. In Singapore, low giant clam density affects reproduction in two ways: 1) it reduces the probability of gametes meeting for fertilisation and 2) individuals are unlikely to reproduce if there are no neighbouring clams to trigger the cascade of spawning synchrony, resulting in component Allee effects on these reduced populations [84].

As giant clams continue to be threatened by anthropogenic activities, active conservation measures are needed [51], [85], [86]. Their sedentary mode of life makes giant clams highly amenable candidates for restocking and stock enhancement [9], [51] and depleted clam populations [10], [57] are currently being restored through these means in Fiji, Palau and the Philippines [9], [30], [58]. However, none of these efforts accounted for whether the transplant sites were effective as source habitats to encourage recruitment in sink sites [87]. The designation of effective restocking sites requires closer examination of metapopulation dynamics, habitat quality and recruitment processes [67], [85] and their potential to augment recruitment [88], [89]. The results from the present study enable the identification and selection of potential source and sink sites for more effective restocking efforts. Metapopulation enhancement can thus be optimised by restocking source populations and subsequently will encourage recruitment in sink populations via larval dispersal [67]. An added strategy to enhance current metapopulations of *T. squamosa* in Singapore waters is to perform *in situ* spawning induction of populations during favourable current periods (e.g. June) to maximise larval retention and settlement.

*Tridacna squamosa* restocking efforts in Singapore are ongoing [2], focusing on *ex situ* breeding and rearing of juvenile clams for out-transplantation. Despite a turbid environment, results from previous outgrowth experiments using imported maricultured juvenile clams were positive [1]. Natural recovery of the *T. squamosa* population in Singapore waters may be possible upon receiving source larvae from nearby offshore coral reefs south of Singapore, but this could take several decades. Even with the potential source larvae, sediment layers on the local reefs continues to be a major challenge for successful settlement and survival of juvenile giant clams in Singapore [2], [90]. The present study supports previous suggestions [3] that the fluted giant clam population in Singapore is experiencing component Allee effects [7], [91], placing constraints on their minimum viable population [92], [93]. Knowledge gaps, such as the critical densities of giant clams required to assure good fertilisation success, have yet to be resolved [57], [86], [94]. Conservation strategies for this species need to account for local hydrodynamics, potential source and sink reef sites, and the (ideally, aggregated) placement of restocked specimens, if the long-term persistence of the population is to be ensured.
